# Leucine Potentiates Glucose-mediated ^18^F-FDG Uptake in Brown Adipose Tissue via β-Adrenergic Activation

**DOI:** 10.3390/biomedicines8060159

**Published:** 2020-06-13

**Authors:** Brenda Huska, Sarah Niccoli, Christopher P. Phenix, Simon J. Lees

**Affiliations:** 1 Biology, Lakehead University, Thunder Bay, ON P7B 5E1, Canada; bhuska@nosm.ca; 2 Medical Sciences, Lakehead University Faculty of Medicine, Thunder Bay, ON P7B 5E1, Canada; saniccol@lakeheadu.ca; 3 Northern Ontario School of Medicine, Medical Sciences Division, Thunder Bay, ON P7B 5E1, Canada; 4 Chemistry, University of Saskatchewan, Saskatoon, SK S7N 5C9, Canada; 5 Thunder Bay Regional Health Research Institute, Thunder Bay, ON P7A 7T1, Canada

**Keywords:** positron emission tomography, non-shivering thermogenesis, diet-induced thermogenesis, glucose metabolism, obesity, propranolol, interscapular brown adipose tissue, UCP-1

## Abstract

Significant depots of brown adipose tissue (BAT) have been identified in many adult humans through positron emission tomography (PET), with the amount of BAT being inversely correlated with obesity. As dietary activation of BAT has implications for whole body glucose metabolism, leucine was used in the present study to determine its ability to promote BAT activation resulting in increased glucose uptake. In order to assess this, 2-deoxy-2-(fluorine-18)fluoro-d-glucose (^18^F-FDG) uptake was measured in C57BL/6 mice using microPET after treatment with leucine, glucose, or both in interscapular BAT (IBAT). Pretreatment with propranolol (PRP) was used to determine the role of β-adrenergic activation in glucose and leucine-mediated ^18^F-FDG uptake. Analysis of maximum standardized uptake values (SUV_MAX_) determined that glucose administration increased ^18^F-FDG uptake in IBAT by 25.3%. While leucine did not promote ^18^F-FDG uptake alone, it did potentiate glucose-mediated ^18^F-FDG uptake, increasing ^18^F-FDG uptake in IBAT by 22.5%, compared to glucose alone. Pretreatment with PRP prevented the increase in IBAT ^18^F-FDG uptake following the combination of glucose and leucine administration. These data suggest that leucine is effective in promoting BAT ^18^F-FDG uptake through β-adrenergic activation in combination with glucose.

## 1. Introduction

Diabetes continues to climb the list of the top ten causes of death world wide [1], placing an ever-increasing personal and financial burden on society. The 2015 International Diabetes Federation Diabetes Atlas states that type 2 diabetes (T2D) is the most prevalent form of the disease [2], with up to 91% of adults with diabetes in high-income countries presenting with this type. Despite developments in prevention of T2D, the number of affected adults continues to increase, with even more adults suffering from impaired glucose tolerance putting them at a high risk of developing the disease in the future. There is, therefore, a growing need for treatment strategies to address this disease. Brown adipose tissue (BAT), once thought to only be relevant in human infants for promoting thermogenesis, has more recently been identified in adults as having the ability to promote whole body glucose metabolism [3].

Substantial BAT depots have been identified in adults using positron emission tomography (PET), with the probability of detection being inversely correlated with body mass index [4]. The metabolic significance of BAT goes beyond thermogenesis. Recently, BAT has been linked to promoting whole body glucose tolerance in an animal model of obesity through endocrine function [5]. Therefore, a better understanding of BAT activation has garnered significant attention in the fields of obesity management and improved glucose metabolism [6,7,8].

BAT is a mitochondria-rich tissue that is primarily responsible for non-shivering thermogenesis. Electron transport is uncoupled from oxidative phosphorylation; this results in energy dissipated as heat [9,10]. This process, also known as mitochondrial proton leak, is able to occur due to the presence of uncoupling protein one (UCP-1) within the cristae of the mitochondria and passive leak of protons across the inner mitochondrial membrane [9,11]. As a consequence, glucose and lipids can be brought into the cell in order to be metabolized without the generation of ATP. This activation process can be stimulated by food intake, known as diet-induced thermogenesis (DIT). Previous research has shown that mixed meals promote BAT glucose uptake [12] and low protein/high carbohydrate meals promote BAT activity [13,14,15,16].

While meals promote BAT activity and thermogenesis, the specific role of leucine on BAT activity is currently unknown. Leucine, which is an essential branched chain amino acid has been linked to the regulation of many metabolic pathways. Chronic leucine supplementation has been shown to promote UCP-1 protein expression in BAT [17] and decrease body weight and fat mass in diet induced obese and high-fat/cholesterol diet mice [17,18]. In addition, this was associated with increased β3-adrenergic receptors, peroxisome proliferator-activated receptor g coactivator-1α, and fibroblast growth factor 21, which are linked to promoting increased UCP-1 [17]. In contrast, chronic leucine supplementation did not alter BAT UCP-1 expression in a low-fat diet model [19]. Acute administration of leucine has been shown to increase circulating insulin [20] and glucose uptake in skeletal muscle [21,22]. These findings might lead to the conclusion that leucine may promote glucose uptake in BAT via an insulin-mediated pathway. However, DIT is typically associated with the activation of adrenergic signalling [23] which has been shown to have an inhibitory action on insulin-mediated glucose uptake. β3-adrenergic stimulation with various agonists has resulted in inhibition of insulin receptor activation, insulin signaling, and subsequent glucose uptake in both human and rodent adipocytes [24,25,26,27]. Therefore, further research is needed to elucidate the role of leucine-mediated glucose uptake in BAT.

The purpose of this study was to 1) investigate the role of leucine in the regulation of glucose uptake into BAT and 2) identify the role of β-adrenergic signaling responsible for any changes in BAT glucose uptake. Glucose uptake was observed using PET following injection with a radiolabeled glucose analog 2-deoxy-2-(fluorine-18)fluoro-d-glucose (^18^F-FDG), a well-established radiotracer used in diagnostic imaging in animals and humans.

## 2. Results

**Figure 1:**
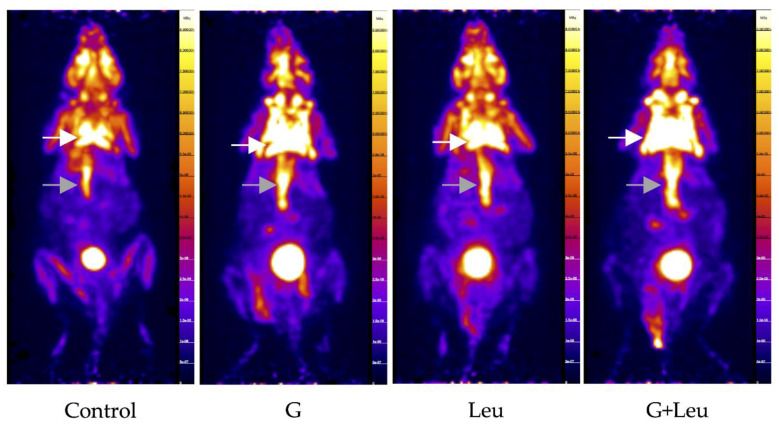
2-deoxy-2-(fluorine-18)fluoro-d-glucose (^18^F-FDG) uptake determined using positron emission tomography (PET) imaging. Representative PET scans for control, glucose (G), leucine (Leu), and the combination of G + Leu-treated mice. The white arrow indicates interscapular brown adipose tissue (IBAT) and the grey arrow indicates paraspinal skeletal muscle.

**Figure 2:**
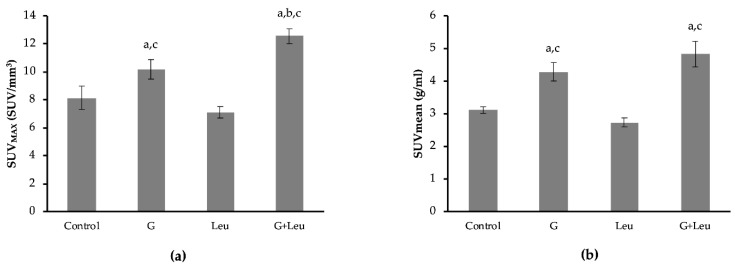
Interscapular brown adipose tissue (IBAT) standardized uptake value (SUV) for control, glucose (G), leucine (Leu), and the combination of G + Leu-treated mice. (**a**) Group mean data for the maximum SUV (SUV_MAX_, units in SUV/mm^3^) was calculated for IBAT for each treatment group. (**b**) Group mean data for the average SUV across the entire tissue (SUVmean, units in g/mL). Data are expressed as mean ± the SEM. A one-way analysis of variance was performed followed by a Fisher LSD post-hoc. a, denotes significant difference compared to control; b, denotes significant difference compared to G (*p* ≤ 0.05); c, denotes significant difference compared to Leu (*p* ≤ 0.05). *n* = 6–8.

In order to determine the ability to stimulate interscapular BAT (IBAT) glucose uptake, 2g/kg glucose was administered with the ^18^F-FDG. As shown in the representative PET scans in Figure 1 IBAT exhibits high basal glucose uptake when the animals are kept at 22 °C. Even with the high basal ^18^F-FDG uptake, glucose administration increased ^18^F-FDG uptake by 25.3% (*p* ≤ 0.05) (Figure 2a,b). When 40 μg/g body weight leucine was administered alone, ^18^F-FDG uptake in IBAT was not different, compared to control (Figure 2a,b). However, when the leucine was administered in combination with the glucose, a 53.6% increase in standardized uptake values (SUV)_MAX_ and 54.8% increase in SUVmean in IBAT were observed compared to control (Figure 2a,b). Interestingly, ^18^F-FDG uptake was 22.5% higher, analyzed as SUV_MAX_, for leucine and glucose combined, compared to glucose alone (*p* ≤ 0.05) (Figure 2a). We also analyzed ^18^F-FDG uptake in cardiac muscle (heart) and skeletal muscle (paraspinal skeletal muscle). Interestingly, no significant changes in ^18^F-FDG uptake were observed for any of the treatments (Table 1).

It was hypothesized that leucine, being an essential branched-chain amino acid, would promote IBAT ^18^F-FDG uptake. However, it was also hypothesized that not all amino acids would have this effect. In order to determine specificity, in a separate cohort of mice, 45 μg/g bodyweight of L-glutamic acid was administered with 2g/kg glucose. As expected, no increase in ^18^F-FDG uptake in IBAT was observed under these conditions (Table 2). Similarly, no differences were observed in the heart and paraspinal skeletal muscle (Table 2).

**Figure 3:**
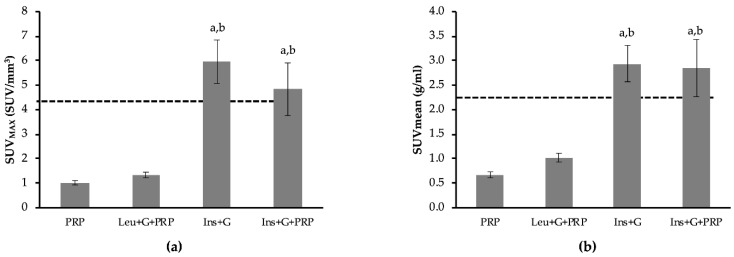
The effect of inhibiting β-adrenergic activation of interscapular brown adipose tissue (IBAT) on 2-deoxy-2-(fluorine-18)fluoro-d-glucose (^18^F-FDG) uptake. β-adrenergic activation was inhibited by pretreatment with propranolol (PRP). Dashed line represents SUV_MAX_ for control/untreated animals. Pretreatment with PRP reduced ^18^F-FDG uptake to 23.6% of control. Following pretreatment with PRP, mice were either untreated (PRP), or treated with leucine and glucose (Leu + G + PRP) or insulin and glucose (Ins + G + PRP). SUV is also reported for insulin and glucose together, without PRP (Ins + G). (**a**) Group mean data for the maximum SUV (SUV_MAX_, units in SUV/mm^3^) was calculated for IBAT for each treatment group. (**b**) Group mean data for the average SUV across the entire tissue (SUVmean, units in g/mL). Data are expressed as mean ± the SEM. A one-way analysis of variance was performed followed by a Fisher LSD post-hoc. a, denotes significant difference compared to PRP; b, denotes significant difference compared to Leu+G+PRP (*p* ≤ 0.05). *n* = 7 for PRP and Leu+G+PRP. *n* = 3 for Ins+G and Ins+G+PRP. *n* = 6–7 for PRP and Leu+G+PRP. *n* = 3 for Ins+G and Ins+G+PRP.

**Figure 4:**
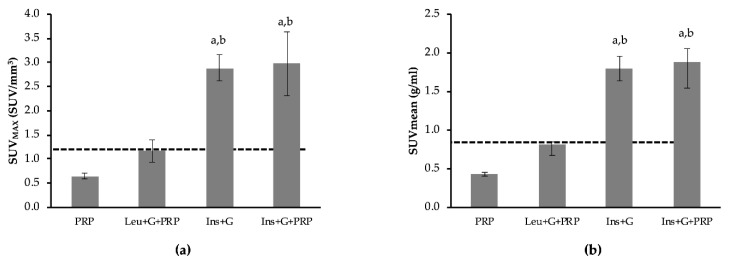
The effect of inhibiting β-adrenergic activation on 2-deoxy-2-(fluorine-18)fluoro-d-glucose (^18^F-FDG) uptake in the heart. β-adrenergic activation was inhibited by pretreatment with propranolol (PRP). Dashed line represents SUV_MAX_ for control/untreated animals. Following pretreatment with PRP, mice were either untreated (PRP), or treated with leucine and glucose (Leu + G + PRP) or insulin and glucose (Ins + G + PRP). SUV is also reported for insulin and glucose together, without PRP (Ins + G). (**a**) Group mean data for the maximum SUV (SUV_MAX_, units in SUV/mm^3^) was calculated for the heart for each treatment group. (**b**) Group mean data for the average SUV across the entire tissue (SUVmean, units in g/mL). Data are expressed as mean ± the SEM. A one-way analysis of variance was performed followed by a Fisher LSD post-hoc. a, denotes significant difference compared to PRP; b, denotes significant difference compared to Leu+G+PRP (*p* ≤ 0.05). *n* = 3–4, if tissue was not visible in scan image, it was excluded.

**Figure 5:**
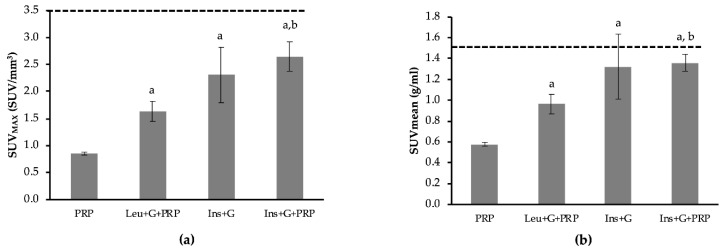
The effect of inhibiting β-adrenergic activation on 2-deoxy-2-(fluorine-18)fluoro-d-glucose (^18^F-FDG) uptake in paraspinal skeletal muscle. β-adrenergic activation was inhibited by pretreatment with propranolol (PRP). Dashed line represents SUV_MAX_ for control/untreated animals. Following pretreatment with PRP, mice were either untreated (PRP), or treated with leucine and glucose (Leu + G + PRP) or insulin and glucose (Ins + G + PRP). SUV is also reported for insulin and glucose together, without PRP (Ins + G). (**a**) Group mean data for the maximum SUV (SUV_MAX_, units in SUV/mm^3^) was calculated for paraspinal skeletal muscle for each treatment group. (**b**) Group mean data for the average SUV across the entire tissue (SUVmean, units in g/mL). Data are expressed as mean ± the SEM. A one-way analysis of variance was performed followed by a Fisher LSD post-hoc. a, denotes significant difference compared to PRP; b, denotes significant difference compared to Leu+G+PRP (*p* ≤ 0.05). *n* = 7 for PRP and Leu+G+PRP. *n* = 3 for Ins+G and Ins+G+PRP.

Various studies have determined that IBAT is active when its thermogenic function is stimulated either through adrenergic stimulation or other routes, and this activation is shown as an increase in ^18^F-FDG uptake [9,28,29,30]. Therefore, in order to determine if the observed stimulation of ^18^F-FDG uptake due to leucine and glucose was acting through β-adrenergic signaling, the beta blocker propranolol (PRP) was used. Following pretreatment with PRP, the combination of leucine and glucose no longer demonstrated a significant increase in IBAT ^18^F-FDG uptake, compared to PRP alone (Figure 3). The results were similar for both SUV_MAX_ (Figure 3a) and SUVmean (Figure 3b) ^18^F-FDG uptake. These data indicate that the combination of leucine and glucose may promote ^18^F-FDG uptake in IBAT via β-adrenergic signaling. As a positive control, the combination of insulin and glucose was used. Insulin and glucose, either with or without pretreatment with PRP, increased IBAT ^18^F-FDG uptake compared to PRP alone (Figure 4a,b) (*p* < 0.05). Interesting results were observed with the heart and paraspinal skeletal muscle in the PRP experiments. In the heart, a non-significant 79.2% increase in SUV_MAX_ and 92.1% increase in SUVmean were observed due to leucine and glucose, compared to PRP alone (Figure 4a,b). This is in contrast to a 6% decrease without inhibition of β-adrenergic signaling (Table 1). Similar to IBAT, insulin and glucose caused significant increases in ^18^F-FDG uptake following pretreatment with PRP in the heart (Figure 4a,b) (*p* < 0.05). Importantly, pretreatment with PRP resulted in a large decrease in paraspinal skeletal muscle ^18^F-FDG uptake, compared to control (represented as dashed line) (Figure 5). Following pretreatment with PRP, SUV_MAX_ increased 66.4% and SUVmean increased 92.2% in response to leucine and glucose in paraspinal skeletal muscle, compared to PRP alone (Figure 5a,b) (*p* < 0.05). In addition, insulin and glucose resulted in a >3-fold increase in ^18^F-FDG uptake, compared to PRP alone (Figure 5a,b) (*p* < 0.05). These data indicate that inhibiting β-adrenergic signaling improves the ability to detect insulin-mediated ^18^F-FDG uptake with PET imaging.

## 3. Discussion

With the emerging importance of BAT as a regulator of whole-body glucose metabolism, a better understanding of dietary BAT activation is required. In the present study, we sought to determine the specific regulatory action of leucine, an essential branched-chain amino acid, which has been linked to many metabolic pathways [17,18,19,20,21,22,31,32]. Our results indicate that leucine alone does not alter IBAT ^18^F-FDG uptake. However, to our knowledge, we are the first to demonstrate that leucine potentiates glucose-mediated IBAT ^18^F-FDG uptake.

While our findings demonstrate that leucine does potentiate the effect of glucose on IBAT ^18^F-FDG uptake, leucine alone showed no effect. These results were not entirely expected, based on previous findings. Diets enriched with leucine have promoted improved whole-body glucose metabolism [32]. Moreover, orally administered branched-chain amino acids can augment the secretion of insulin via the action of incretins, and could further stimulate glucose uptake [33], while leucine specifically has been shown to increase circulating insulin [20] and glucose uptake in skeletal muscle [21,22]. One possible explanation for our results may be the magnitude of the insulin response following the dose of leucine administration. We chose to use a dose equivalent to 0.3 mmol/kg body weight (40 μg/g body weight). This dose was chosen based on the results of Poncet et al. [34], demonstrating that while 40 μg/g body weight leucine was a submaximal dose, it did result in an approximately 2-fold increase in plasma leucine and an increased pS6K (T389)/S6K ratio in skeletal muscle. However, other studies have used higher doses of leucine (e.g., 3 mmol/kg body weight) or infusions of various amino acids that result in higher circulating concentrations of leucine [20,21]. Garlick and Grant [21] utilized glucose infusion in combination with amino acids in order to demonstrate increased skeletal muscle protein synthesis. Our results are in agreement with Garlick and Grant [21], in that the combination of glucose with leucine did promote a response at the tissue level.

Not all amino acids have been shown to alter circulating insulin and/or glucagon levels. For example, it has been previously demonstrated that while leucine infusion increases circulating insulin, l-glutamic acid infusion did not cause changes in insulin or glucagon levels [20]. In the present study, l-glutamic acid was administered with the addition of glucose to test this selective response on IBAT ^18^F-FDG uptake. It was determined that L-glutamic acid did not have the same effect as leucine on ^18^F-FDG uptake in IBAT under hyperglycaemic conditions (Table 2). These results indicate that amino acid action on IBAT ^18^F-FDG uptake does demonstrate specificity to certain amino acids.

The mechanism by which leucine potentiates glucose-mediated IBAT ^18^F-FDG uptake is not certain. In the present study, pretreatment with PRP prevented the effect of glucose and leucine on promoting IBAT ^18^F-FDG uptake. Propranolol is a nonselective β-adrenoreceptor antagonist that is clinically used to target noradrenergic systems and block β-adrenoreceptors in the central nervous system to treat various ailments [35,36]. Propranolol acts with high affinity to β1 and β2 adrenoreceptors, and lower affinity to β3-adrenoreceptors [37] and has been shown in humans to decrease ^18^F-FDG uptake in the BAT of clinical patients receiving PET scans [38,39]. In rodents, IBAT is known to have all three subtypes of β-adrenoreceptors [40], with the β1-adrenergic signaling pathway mediating a significant portion of sympathetic nervous system stimulation of adaptive thermogenesis [41], and the β3-receptor involved in energy expenditure and insulin sensitivity (reviewed in [30]). The present study data indicate that glucose and leucine regulate IBAT ^18^F-FDG uptake via β-adrenergic activation, and future studies will be required to establish which of the β-adrenoreceptors are responsible for these observed effects. Previous work does support the interpretation of macronutrient stimulation of the β-adrenergic activation of BAT. Following fasting, glycogen accumulation in IBAT following re-feeding is robust (~2000-fold compared to fasted state) [12]. However, this response is inhibited by Alpha-Methyl-Para-Tyrosine [12], which has been shown to effectively reduce epinephrine and norepinephrine in BAT fat pads [42,43,44]. Interestingly, leucine deprivation has also been shown to decrease fat mass and increase both UCP-1 expression and activation of the sympathetic nervous system [45,46]. Taken together, our results suggest a role of the central nervous system in promoting increased glucose uptake into BAT.

While it is difficult to know the mechanism by which leucine potentiates glucose-mediated IBAT ^18^F-FDG uptake, recent evidence highlights a possible role of the nutrient-sensitive kinase mTORC1. Amino acids, glucose and insulin have been shown to promote the activation of mTORC1 [47] by the Ras-related GTPases (Rags) and the Ras homolog enriched in the brain (Rheb) [48]. Since mTORC1 activity in the hypothalamus has been demonstrated to drive sympathetic nerve activity [49,50,51], it is possible that glucose and the combination of glucose and leucine increased mTORC1 activity in the hypothalamus, leading to increased sympathetic activation and IBAT glucose uptake. Alternatively, leucine may potentiate β-adrenergic activation in an mTORC1-independent manner. β-adrenergic receptor activation in IBAT increases cyclic adenosine monophosphate (cAMP), leading to protein kinase A (PKA)-dependent activation of metabolism [52]. It is possible that elevated leucine may enhance signaling via the cAMP/PKA pathway or cause increased UCP-1 activation. While the effects of leucine on UCP-1 activity are currently unknown, there is evidence that several other dietary modulators of BAT thermogenesis exist [53]. Future work to measure downstream signaling effects from the combination treatment is necessary to better understand the mechanism regulating the improvement on ^18^F-FDG uptake.

In the absence of PRP, glucose administration did not increase ^18^F-FDG uptake in the heart or paraspinal skeletal muscle (Table 1). However, this might not be unexpected due to conditions used for imaging. For example, the temperature of the room was 22 °C ± 1 °C, which is below thermoneutral, and the animals were awake during the uptake period. Both of these would contribute to increased sympathetic nerve activity [28,54]. It is known that β agonists can increase skeletal muscle glucose utilization [55] and uptake [56], promote insulin secretion and alter glycogenolysis and glucose release from the liver [56]. However, following the inhibition of β-adrenergic signalling (PRP pretreatment), the combination of glucose and leucine caused a non-significant 79–92% increase in ^18^F-FDG uptake in the heart (SUV_MAX_ and SUVmean; Figure 4a,b). With PRP preteatment, insulin administration, used as a positive control, demonstrated a nearly 3-fold increase in cardiac muscle ^18^F-FDG uptake (Figure 4). In addition, Figure 5 demonstrates that leucine and glucose administration did increase ^18^F-FDG uptake in paraspinal skeletal muscle when mice were pretreated with PRP (66–92% for SUV_MAX_ and SUVmean). These findings suggest that even though care was placed on limiting animal stress during PET imaging, there is an adrenergic response that can affect glucose metabolism and mask treatment effects. Future studies aimed at utilizing PET imaging of insulin-mediated glucose metabolism might benefit from using PRP pretreatment to minimize the effect of β-adrenergic activation.

## 4. Materials and Methods

### 4.1. Animals

All animal experiments were approved by and performed in accordance with the institutional animal care committee guidelines at Lakehead University (Animal Use Protocol #10 2014, file #1463841, approved 20 May 2014). The animals were cared for in accordance with the Guide to the Care and Use of Experimental Animals available from the Canadian Council on Animal Care. C57BL/6 male mice were obtained from Charles River Laboratories Inc. (Saint-Constant, QC, Canada) at 6–8 weeks of age. Mice were housed in Innovive disposable cages (Innocage^®^, San Diego, CA, USA) with the pre-filled bedding replaced with All Living Things^®^ Small Pet Bedding (Phoenix, AZ, USA). A small amount of bedding from the mouse transport box was added to cages to help with adjustment. Mice had constant water access and were fed ad libitum unless otherwise stated. Food was LabDiet 5001 Rodent Diet (Land of Lakes Inc., Arden Hills, MN, USA). Mice were housed in Innovive Innorack^®^ IVC (San Diego, CA, USA) mouse racks. Enrichment was provided with plastic enrichment domes in each cage. Mice were acclimatized after arrival for at least one week before experiments began. Housing temperature and humidity levels were controlled and kept at 22 °C ± 3 °C and 50% ± 9%, respectively. Mice were kept on a 12 h light/dark cycle.

### 4.2. In Vivo Imaging with PET

At least five days prior to experiments, mice were individually housed, with a handful of bedding from the original group housed cages added to the individual cage to help with adjustment. Five to seven hours prior to experimentation, mice were placed into clean cages with no food for the fasting period, but free access to water. Mice were transported to the experimentation room, where a constant temperature was maintained (22 °C ± 1 °C) for the course of the experiments. ^18^F-FDG (Center for Probe Development and Commercialization, Hamilton, ON, Canada) was injected into the intraperitoneal cavity of anaesthetized mice at a targeted dose of approximately 20 μCi. The actual dose for each injection was calculated based on dose calibrator measurements of the syringe pre- and post-injection. All other treatments were also given through an intraperitoneal injection. One hour after treatment injection, mice were anesthetized with 2% isoflurane again for three min. Mice were then placed in the imaging chamber in prone position and inserted into the G4 PET/X-Ray scanner (Sofie Biosciences, Culver City, CA, USA). The imaging chamber contains a nose cone for isoflurane administration (held at about 1.5%) and the bed was heated to 37 °C. Image acquisition proceeded for ten min. After the scan was completed, an X-ray was performed as per the specifications of the G4 scanner. Following imaging, mice were returned to fasting cages to recover from anesthesia prior to being returned to original cages containing food. VivoQuant™ (Version 1.23, invicro, Boston, MA, USA) image analysis software was used to reconstruct and quantify ^18^F-FDG uptake values within specific tissues. Tissues analyzed were heart, paraspinal skeletal muscle, and interscapular brown adipose tissue (IBAT). A volume of the entire tissue (region of interest) was drawn using a spherical draw tool. Once the entire tissue was encompassed, the average standardized uptake values (SUVmean, units in g/mL) and maximum standardized uptake values (SUV_MAX_, units in SUV/mm^3^) were calculated. SUVmean reports the average radiotracer concentration within a user-defined region of interest. Although some debate still exists in the literature, issues of reproducibility when using SUVmean to capture ^18^F-FDG avidity can result especially in tissues that are difficult to spatially define like BAT. SUV_MAX_, which is the maximum radiotracer concentration within a user-defined region, is considered more reproducible than SUVmean since the highest ^18^F-FDG avidity within a voxel inside a region of interest is less influenced by interobserver variability [57,58]. Animal body weight and exact ^18^F-FDG dose were taken into account for this analysis.

### 4.3. Treatments

In order to assess the regulation of IBAT glucose uptake and signaling, the following treatments were administered in addition to ^18^F-FDG. Glucose and amino acid treatments included 2 g/kg bodyweight glucose (Fisher, Whitby, ON, Canada), 40 μg/g bodyweight l-leucine (Sigma Aldrich, Oakville, ON, Canada), and the combination of 2 g/kg bodyweight glucose and 40 μg/g bodyweight l-leucine. In a separate cohort of mice, the combination of 2 g/kg bodyweight glucose and 45 μg/g bodyweight l-glutamic acid (Sigma Life Science, St. Louis, MO, USA) was also tested. The leucine dose was chosen based on a previous report demonstrating an approximately 2-fold increase in plasma leucine and an increased pS6K (T389)/S6K ratio in skeletal muscle [34]. In another separate cohort of mice, inhibition of β-adrenergic action using an additional injection of 150 μg/mouse of propranolol hydrochloride (PRP) (Sigma-Aldrich, St. Louis, MO, USA) 30 min before any other treatment was used. In order to verify insulin-mediated effects on insulin sensitive tissues, 10 U/kg insulin (Humulin R; Eli Lilly, Toronto, ON, Canada) was used in combination with 2 g/kg glucose. All treatments were made in sterile water. The studies performed on different cohorts of mice were done during different times of year. Seasonal effects are the likely cause of differences between the studies, which is not an uncommon observation in metabolism research. Importantly, we always had separate control groups in the different cohorts for our studies to account for these seasonal effects.

### 4.4. Statistics

For PET imaging, outliers were identified as greater than 1.5 times the interquartile distance above the third quartile or less than 1.5 times the interquartile distance below the first quartile, which is the standard SPSS outlier definition. Significance was determined for all data using a one-way analysis of variance with a Fisher LSD post-hoc. Data are presented as means ± standard error of the mean (SEM). Significance was accepted at *p* ≤ 0.05.

## 5. Conclusions

Dietary activation of BAT may have implications for promoting improved whole-body glucose metabolism. An improved understanding of the mechanisms responsible for increasing BAT activity may be useful for prevention and/or treatment of metabolic syndrome and T2D. Here, we demonstrate that leucine can potentiate glucose-induced ^18^F-FDG uptake in IBAT. Our results indicate that the effect of leucine is mediated through β-adrenergic activation of IBAT. This finding is important because the stimulation of BAT ^18^F-FDG uptake with leucine may be effective in insulin-resistant states. Future research could be aimed at determining whether the effects observed with glucose and leucine occur during cold stimulation and thermoneutral conditions, and to determine if leucine promotes glucose-mediated activation of BAT in models of obesity and T2D.

## Figures and Tables

**Table 1:** Glucose uptake following glucose (G), leucine (Leu), or the combination of G + Leu in cardiac and skeletal muscle.

Tissue	Treatment	SUV_MAX_	±	SEM
Heart	Control	1.80	±	0.23
	G	1.59	±	0.04
	Leu	1.46	±	0.14
	G + Leu	1.69	±	0.05
Paraspinal skeletal muscle	Control	4.69	±	0.42
	G	4.25	±	0.45
	Leu	4.68	±	0.68
	G + Leu	4.19	±	0.43

**Table 2:** Glucose uptake following glutamic acid (Glu) and glucose (G) injection.

Tissue	Treatment	SUV_MAX_	±	SEM
Interscapular brown adipose tissue	Control	12.07	±	1.30
	Glu+G	11.61	±	1.20
Heart	Control	2.33	±	0.50
	Glu+G	1.99	±	0.18
Paraspinal skeletal muscle	Control	6.06	±	0.96
	Glu+G	5.13	±	0.77

## Abbreviations

^18^F-FDG T2D	2-deoxy-2-(fluorine-18)fluoro-d-glucose Type 2 diabetes
BAT	Brown adipose tissue
PET	Positron emission tomography
UCP-1	Uncoupling protein one
DIT	Diet-induced thermogenesis
IBAT	Interscapular brown adipose tissue
SUVmean	Average standard uptake values
SUV_MAX_	Maximum standard uptake values
PRP	Propanolol hydrochloride
SEM	Standard error of the mean
Rags	Ras-related GTPase
Rheb	Ras homolog enriched in the brain
PKA	Protein kinase A
